# Tne Condition of Vision the Best Test of the Squinting Eye

**Published:** 1856-08

**Authors:** C. Holthouse


*The Condition of Vision the Best Test of the Squinting Eye.* By C. Holthouse, Esq.

It is remarkable, that among the numerous writers on the subject of strabismus there should be so little accordance as to the best test for discovering the faulty eye in those cases in which the squint shifts from one to the other. To be convinced of this, one has only to refer to the works of the three latest English writers on the subject. Walton observes, "It is not an easy matter to determine which is the defective eye, and the sound eye is sometimes operated on. When this cannot be readily ascertained, I place the patient in front of me, at a distance of two or three yards, and direct him to cover one eye,—say the left,—and look at me with the other, keeping the head straight: the right eye will be in the centre of the orbit; I then direct him to uncover the left. Now, if the right, which has not been closed, is normal, it will keep its central position, while the left eye is turned inwards; but if it be deformed it will turn in, while the left will become straight. The experiment should be reversed." Again: "When a patient is under examination, he is generally excited, and exerts the orbital muscles unnaturally; and then it is out of the question to obtain a sight of the squinting eye even in a moderately quiescent state; and hence I have sometimes been obliged to wait until a second visit to detect the faulty eye." Mackenzie follows Lucas's rule for discovering whether one or both eyes are effected. "We are able readily to detect non-alternating as well as alternating strabismus, by desiring the patient to look steadily with either of his eyes at any object straight before him, while with one hand we hide the object from his other eye, but keep the hand sufficiently raised towards the temple to allow us to watch the movements of the eye which is thus shaded. Whether the strabismus is alternating or non-alternating, the shaded eye is distorted. If in such a case we close both eyes, and then suddenly raise the upper lid of either while the other remains closed, the one which is opened is seen to be distorted. If both eyes are suddenly opened, the pupil of the worse eye is discovered to be more distorted than that of the better eye. If, on trying these experiments, the eye which is shaded, or either of them, on being opened suddenly showed no obliquity, we would pronounce the eye to be sound."

Dixon observes, "When both eyes appear to be effected with strabismus and to turn inwards, it becomes a question which eye ought to be operated on. Various optical tests have been suggested to enable the Surgeon to decide this point; but it usually happens that a patient, when subjected to any of these tests, is so anxious and embarrassed, that he becomes very liable to a sudden increase of strabismus in the eye, which, on ordinary occasions, would be effected in the slighter degree; and from this cause the experiment may fail to infallibly determine the question. I believe the best rule is, to watch attentively which eye squints in the more decided manner, when the patient uses both eyes in his ordinary way, and to operate on that in which the distortion predominates."

In my lectures on strabismus, published two years ago, I pointed out the law which determines the alternation of the squint; and since then I have had abundant opportunities of testing its value, as well as proving the inutility of all other tests. This law may be thus expressed:

—The less the difference in the visual power of the two eyes, the greater the tendency of the squint to alternate; and, conversely, the greater the difference in the visual power of each eye, the less the tendency to shift.

The visual power, then, is the only test that can be depended on, and the rule of practice would appear to be this;—In true alternating squint, where the power of the two eyes is alike, it is immaterial which eye is operated on; while in the false, and by far the most frequent, variety of alternating squint, that eye should be selected for operation the visual power of which is inferior.

Shortly before my return from the East, I was requested by Dr. McCraith, of Smyrna, to operate on a young Greek lady, who squinted very decidedly, though with which eye it was difficult to determine, as it continually shifted from one to the other; it seemed however, to have a preference for the left. Walton's test failed completely; but vision was most perfect in the left; that is, in the eye apparently most affected; it was not bad in either, the patient being able to read with both, though a smaller type with the left than with the right. Notwithstanding the right eye seemed to be the straight one, I operated on it rather than on the other, owing to its less perfect vision. The correctness of the diagnosis was at once made manifest, by the extraordinary size and toughness of the tendon of the internal rectus, as well as by the rectification of the deformity in the other eye. The preference (if I may so use the term) of a squint for the better eye is a curious phenomenon that was noticed many years ago by Dr. Radcliff Hall, and is occasionally due to certain extraneous and sometimes appreciable causes. A little boy, 16 years of age, was brought to the public Dispensary for a stye on the outer part of the left upper lid; the eye on the same side was also considerably inverted. At first sight, I considered that this was a case of single convergent strabismus of the left eye; but on placing a book before it, I found its vision quite perfect; while, on placing it opposite the other, or straight eye, he could with difficulty decipher the letters. This fact at once assured me that the latter was really the strabismic organ, and that the inversion of the opposite one was only temporary. In the course of a few days, the stye had disappeared; and, as I anticipated, the squint had disappeared with it, and had been transferred to the opposite eye. I think there can be little doubt that the inflammatory condition of the eyelid in this case determined the strabismus to the good eye; for the movement of the lids, being in great measure regulated by that of the eye ball, the quiescent condition of the latter in the inner canthus would entail a corresponding condition of the former, and thus the pain arising from their movements be avoided. The bad eye was thus instinctively called into requisition, so that a superficial observer would readily have mistaken it for the good one.—*Medical Times and Gazette.*

